# Dual Analytical Evaluation of Generic Intravenous Vancomycin HCl in Saudi Arabia Using Microbiological and HPLC-DAD Approaches

**DOI:** 10.3390/antibiotics15050470

**Published:** 2026-05-06

**Authors:** Haya S. Alzeer, Amani T. Alsufyani, Mai S. Alwathnani, Afnan Althobaiti, Manal Almusa, Fatimah M. Alamri, Haneen H. Aldossari, Yahya M. Alshehri, Lenah E. Mukhtar, Fahad M. Alreshoodi, Fahad S. Aldawsari

**Affiliations:** 1 Saudi Food and Drug Authority (SFDA), Riyadh 13513, Saudi Arabiaafnan.althob@gmail.com (A.A.); fmreshoodi@sfda.gov.sa (F.M.A.);; 2 Department of Pharmaceutics, College of Pharmacy, Prince Sattam Bin Abdulaziz University (PSAU), Al-Kharj 16273, Saudi Arabia; maialwathnani@gmail.com; 3 Department of Chemistry, College of Science, King Khalid University (KKU), Abha 62529, Saudi Arabia; 4 Department of Chemistry, College of Science, University of Bisha (BU), Bisha 61922, Saudi Arabia; 5 Department of Biochemistry, College of Science, King Saud University (KSU), Riyadh 11451, Saudi Arabia; haneenaaldosari@gmail.com

**Keywords:** vancomycin hydrochloride, generic equivalence, intravenous injection products, therapeutic drug monitoring, agar diffusion, post-market surveillance, analytical chemistry, HPLC (high-performance/pressure liquid chromatography)

## Abstract

Background/Objectives: Vancomycin hydrochloride (HCl) for injection is a vital antibiotic for severe infections. Vancomycin is an indispensable first-line antibiotic for life-threatening infections like methicillin-resistant *Staphylococcus aureus* (MRSA) bacteremia and serves as a key oral agent for severe *Clostridium difficile* infection. This makes rigorous quality monitoring essential. Concerns about the efficacy of certain generic vancomycin HCl products have prompted regulatory agencies to increase post-market evaluations to ensure patient safety. Aligned with these efforts, this study aimed to comparatively evaluate the potency and quality of five randomly procured generic intravenous vancomycin HCl products marketed in Saudi Arabia. Methods: Using both microbiological and chemical methodologies as recommended by the US Pharmacopeia (USP) guidelines. Results: The microbiological assay, utilizing the USP “Antibiotics—Microbial Assay” method using *Bacillus subtilis* ATCC 6633 via the agar diffusion technique, demonstrated a strong linear correlation (R^2^ > 0.98) between inhibition zone diameters and vancomycin concentration. All five tested generics products showed compliance (~ 105%) with USP <81> biological activity standards. For chemical quantification, a High-Performance Liquid Chromatography-Diode Array Detector (HPLC-DAD) method was developed and validated according to ICH Q2(R2) guidance. This method showed excellent linearity (R^2^ = 0.998), accuracy (96.9–102%), and good precision. Conclusions: Both methods confirmed the potency of the tested generics within USP limits. However, minor variations observed between microbiological and chemical results highlight the importance of employing complementary techniques for comprehensive quality assessment. These findings underscore the importance of robust regulatory frameworks and ongoing post-market surveillance to ensure the continued safety, efficacy, and availability of quality-assured generic antibiotics in the Saudi market.

## 1. Introduction

**Figure 1:**
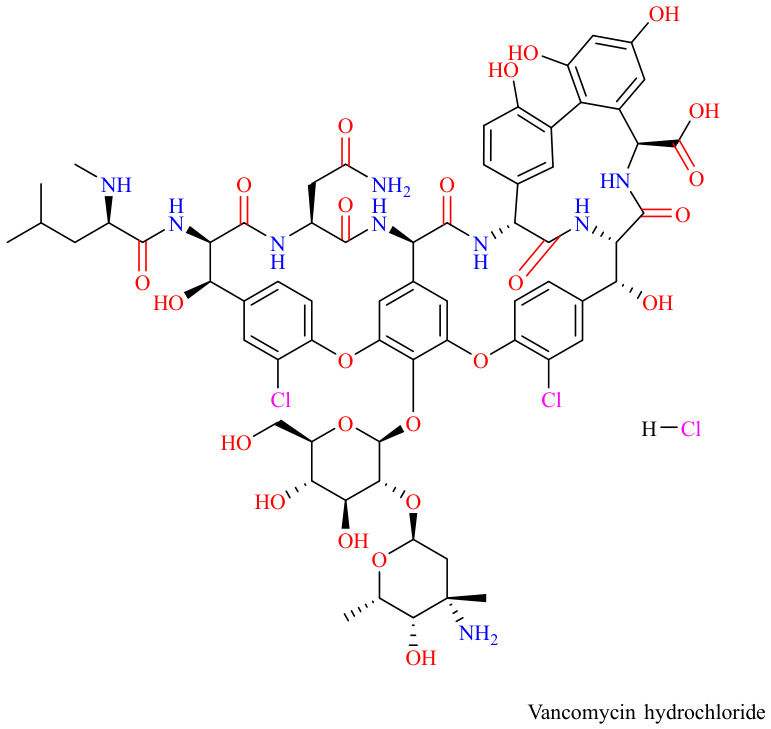
Chemical structure of vancomycin hydrochloride. The molecular formula is C_66_H_76_Cl_3_N_9_O_24_. The Chemical structure was created using ChemDraw, version: 20.0.0.41. The molecular formula was derived based on PubChem data (PubChem 2.2 release 2024).

Vancomycin, commonly formulated as vancomycin hydrochloride (HCl) for intravenous (IV) administration, is a critical glycopeptide antibiotic widely used to treat severe infections caused by Gram-positive bacteria, including methicillin-resistant *Staphylococcus aureus* (MRSA). For *Clostridium difficile* infection (CDI), however, vancomycin is given orally due to its limited ability to penetrate the gut mucosa [1,2]. As illustrated in (Figure 1) vancomycin HCl (C_66_H_76_C_l3_N_9_O_24_), the hydrochloride salt form of vancomycin exhibits markedly superior aqueous solubility compared to the free base. This enhanced solubility facilitates faster and consistent dissolution, ensuring reliable formulation performance and effective intravenous administration, which is essential for managing life-threatening infections [3]. Vancomycin HCl exerts its antibacterial activity by inhibiting bacterial cell wall synthesis through binding to the D-Ala-D-Ala terminus of peptidoglycan precursors and ultimately causing cell death [3,4,5]. Vancomycin HCl is available as a powder for injection in 250 mg, 500 mg, and 1 g dosages, and is recommended as a first or second-line therapy for serious conditions such as endophthalmitis, necrotizing fasciitis, and high-risk febrile neutropenia [6].

Originally isolated in the 1950s from *Amycolatopsis orientalis* in a Borneo soil sample [7], vancomycin has evolved from a novel discovery into a cornerstone agent for combating multidrug-resistant (MDR) Gram-positive pathogens, particularly methicillin-resistant *Staphylococcus aureus* (MRSA) [8,9]. First approved in 1958 to combat penicillin-resistant *Staphylococcus aureus*, its importance surged in the 1980s as it became the last-line defense against the rising threat of methicillin-resistant *S. aureus* (MRSA) [8,10]. However, this reliance was profoundly challenged by the emergence of reduced susceptibility in the late 1990s [11], culminating in the first confirmed infection caused by vancomycin-resistant *S. aureus* (VRSA) in the United States by 2002 [12]. Despite these resistance challenges and the development of newer agents, vancomycin remains an indispensable therapeutic option for serious MDR infections, underpinning its enduring critical status in the antimicrobial arsenal [13,14]. This status is reflected in its classification under the World Health Organization’s (WHO) “Watch” category of the AWaRe framework, emphasizing the need for careful stewardship and strict quality assurance to preserve its effectiveness in the management of life-threatening infections [6].

Its sustained role as a last-line defense underscores the paramount importance of ensuring the consistent quality, potency, and reliability of every dose administered, especially as it is now widely available in generic formulations [8]. While the quality, safety, and efficacy of vancomycin HCl are stringently regulated by national and international health authorities, global concerns persist regarding the potency and therapeutic reliability of certain generic formulations. These concerns are substantiated by reports of treatment failures and adverse outcomes [15].

Persistent global concerns regarding the quality and efficacy of generic vancomycin products have catalyzed increased regulatory scrutiny. A foundational concern arose from studies demonstrating that certain generic formulations, despite meeting standard pharmaceutical equivalence and bioequivalence criteria, failed to achieve comparable clinical outcomes to the innovator product in vivo, suggesting limitations in traditional equivalence paradigms for this critical antibiotic [16,17]. These findings of potential efficacy gaps have been compounded by recurring quality control failures in the post-market phase, exemplified by recalls such as the 2024 FDA action against an orally administered vancomycin product due to superpotency, which poses a direct toxicity risk to patients. This landscape of concern exists against the enduring backdrop of advancing bacterial resistance, notably marked by the first report of vancomycin-resistant *Staphylococcus aureus* (VRSA) in 2002. Collectively, these factors underscore why generic vancomycin remains under intense international surveillance and highlight the imperative for robust, multifaceted quality assessment strategies beyond initial approval metrics to ensure patient safety and therapeutic reliability.

Such failures can be traced to variability in critical areas, such as raw material sourcing, manufacturing processes, and analytical methodologies, which directly impact essential pharmaceutical attributes like potency, dissolution, and bioavailability [18]. Failure to meet regulatory potency standards can result in subtherapeutic drug levels, leading to infection progression and the development of antimicrobial resistance, particularly with organisms like MRSA, and may even result in mortality [19,20,21,22]. Economically, this also contributes to increased healthcare costs. Moreover, improper dosing raises the risk of toxicity, including nephrotoxicity and ototoxicity [20,21,22]. Therefore, accurately determining the optimal vancomycin dosage is crucial to achieving effective therapeutic outcomes while minimizing adverse effects [19,20,21,22,23,24].

Globally, the vancomycin HCl market holds significant economic importance due to its role in combating life-threatening infections. The growing demand is driven by rising antibiotic resistance, positioning vancomycin as a strategic player in the global antibiotic sector. Market expansion is driven by the emergence of multidrug-resistant (MDR) bacterial strains and the urgent need for effective therapies [25]. North America leads this market due to advanced healthcare infrastructure, while Europe contributes through strict regulatory controls and sustained clinical need [26]. In the Asia-Pacific region, countries such as India are experiencing rapid growth due to rising healthcare investments and awareness of antimicrobial resistance [26]. In the future, the vancomycin HCl market is expected to continue expanding significantly from 2023 to 2031, as emerging markets further develop their healthcare infrastructure and investment in antimicrobial resistance management increases [25].

Consequently, global regulatory agencies including the U.S. Food and Drug Administration (FDA), European Medicines Agency (EMA), and the Saudi Food and Drug Authority (SFDA) have increasingly prioritized the evaluation of generic drugs [10,27,28,29,30]. These efforts underscore the necessity for thorough pre- and post-market evaluations to ensure public health is protected and trust in generics is maintained. In line with these international efforts, the FDA oversees vancomycin HCl approval through clinical trials, bioequivalence studies, and post-marketing surveillance (PMS) [31,32,33]. Additionally, the United States Pharmacopeia (USP) and British Pharmacopoeia established the standards for the potency and quality of pharmaceuticals in general and on the “vancomycin HCl” in a specific topic, which manufacturers must meet to ensure medication safety and efficacy [34,35]. Strict adherence to these standards helps prevent issues related to substandard or contaminated products, thereby protecting public health.

In Saudi Arabia, SFDA establishes and enforces a comprehensive regulatory framework governing the registration, variation, and renewal of pharmaceutical products, including the market authorization application (MAA) [29]. This framework is designed to promote effective collaboration between stakeholders throughout the product authorization lifecycle. Furthermore, the SFDA offers a Breakthrough Medicine Program that facilitates the expedited review of essential medications for life-threatening conditions by fostering early collaboration between regulators and health developers [18]. Within the Saudi market, vancomycin HCl is available in generic formulations, improving accessibility and affordability while remaining subject to stringent regulatory standards to ensure quality, safety, and efficacy, ultimately prioritizing patient protection [18,29].

In Saudi Arabia, vancomycin HCl is primarily imported; however, significant government investment under Vision 2030 aims to expand local pharmaceutical manufacturing and reduce reliance on imports and enhance national self-sufficiency [29,36]. Ensuring the potency of locally manufactured vancomycin is crucial for effective treatment, preventing resistance, and meeting regulatory requirements, particularly since the latest statistics indicate an increase in antibiotic consumption globally [25,37]. Regular potency assessments support patient health and ensure the drug’s ongoing efficacy and safety, while simultaneously encouraging local industry growth and ensuring a cost-effective and reliable therapeutic option [29,36].

To evaluate intravenous products of vancomycin HCl, both microbiological assay and the chemical analytical method of high-performance liquid chromatography (HPLC) are employed, as per USP recommendations. However, each technique offers distinct advantages in assessing drug potency and purity [15]. Microbiological assay determines biological activity directly based on vancomycin’s inhibitory effect on susceptible microorganisms [38,39]. In contrast, HPLC allows chemical quantification by separating and identifying the active component and potential impurities or degradation products based on retention time and peak area [15,35].

While global concerns exist, there is a paucity of publicly available, methodologically comprehensive data on the quality of generic vancomycin HCl in the Saudi Arabian market. This study was designed to address the identified gap through a dual-method analytical evaluation of approved generic intravenous vancomycin HCl products commercially available in Saudi Arabia. In accordance with USP recommendations, a dual analytical approach was employed, combining a microbiological assay (cylinder-plate technique) to determine biological potency with a validated HPLC-DAD method for precise chemical quantification. By comparing the results obtained from these orthogonal techniques on identical product samples, a robust assessment of compliance with pharmacopeial standards was performed. The findings generated are intended to furnish critical evidence for regulatory bodies, support ongoing pharmacovigilance efforts, and ultimately ensure the therapeutic reliability of this last-line antibiotic for patients in the region.

## 2. Materials and Methods

### 2.1. Chemicals and Materials

Standard reference material for vancomycin hydrochloride was obtained from the United States Pharmacopeia (Cas No. 1404-93-9, product No. 125407, Rockville, MD, USA). Five distinct generic brands of vancomycin hydrochloride for intravenous injection (samples U_1_–U_5_) were acquired from local pharmacies in Saudi Arabia.

For the chromatographic analysis, acetonitrile, tetrahydrofuran, and triethylamine (all HPLC-grade) were purchased from (Sigma-Aldrich, Darmstadt, Germany). Phosphoric acid (85%, analytical grade) was obtained from (Merck, Darmstadt, Germany). Deionized water was prepared using a Milli-Q^®^ water purification system (Merck Millipore, Burlington, MA, USA).

For the microbiological assay, pancreatic digest of casein and agar were obtained from (Becton, Dickinson and Company, Franklin Lakes, NJ, USA). Sodium chloride, dibasic potassium phosphate, dextrose, and monobasic potassium phosphate (all analytical grade) were purchased from (Sigma-Aldrich, Darmstadt, Germany). Antibiotic Assay Medium No. 32 was prepared according to the standard formulation described in USP <81> Antibiotics—Microbial Assays, consisting of peptone (6.0 g), pancreatic digest of casein (4.0 g), yeast extract (3.0 g), beef extract (1.5 g), manganese sulfate (0.3 g), dextrose (1.0 g), and agar (15.0 g) in 1000 mL purified water (pH after sterilization 6.6 ± 0.1). Antibiotic Assay Medium No. 8 was also prepared according to the standard formulation consisting of peptone (6.0 g), yeast extract (3.0 g), beef extract (1.5 g), and agar (15.0 g) in 1000 mL purified water (pH after sterilization 5.9 ± 0.1). Unless otherwise specified, the medium components were obtained from (Oxoid, Thermo Fisher Scientific, Basingstoke, UK).

General laboratory consumables, including volumetric flasks, analytical pipettes, 0.22 μm nylon syringe filters, beakers, and HPLC vials, were used. For the agar diffusion assay, disposable polystyrene petri dishes (20 mm × 100 mm) with caps were obtained. Stainless steel cylinders (outer diameter: 8 ± 0.1 mm, inner diameter: 6 ± 0.1 mm, height: 10 ± 0.1 mm) were used and thoroughly cleaned prior to use to eliminate any residual contaminants that might compromise the assay findings.

### 2.2. Microbiological Assay

**Figure 2:**
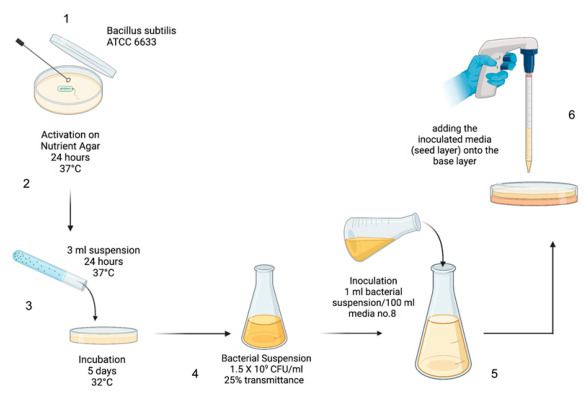
Workflow for the preparation of *Bacillus subtilis* ATCC 6633 suspension for the agar diffusion assay. The workflow illustrates the six key steps, including culture activation, inoculum adjustment, and seed layer preparation, following USP <81> guidelines.

**Figure 3:**
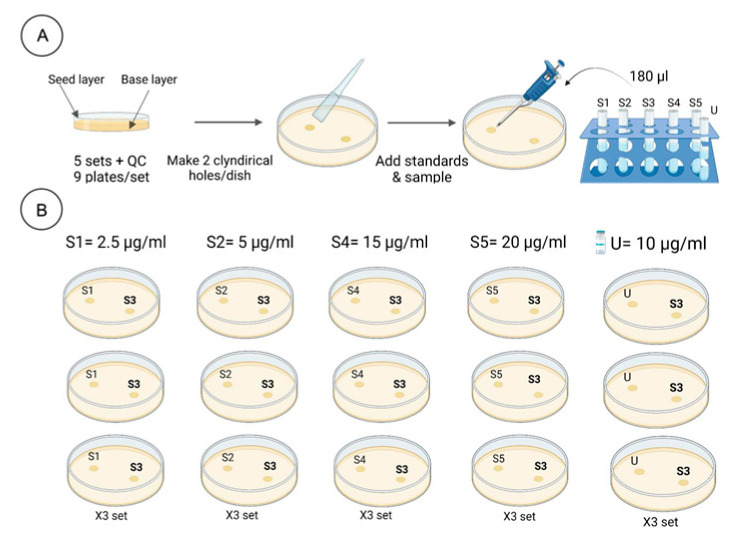
Schematic diagram of the cylinder-plate assay for vancomycin HCl potency testing. The assay involves two main steps: (**A**) preparing agar plates with base and seed layers, and creating two cylindrical wells per plate; and (**B**) the addition of 180 µL of standards (S_1_, S_2_, S_4_, and S_5_) at concentrations of 2.5, 5, 15, and 20 µg/mL, respectively. Vancomycin HCl samples (U_x_) at 10 µg/mL are compared with a reference standard (S_3_, 10 µg/mL) in triplicate sets of plates. Inhibition zones are measured to determine the potency of the test sample relative to the reference standard.

The microbiological test used to evaluate the efficiency of vancomycin HCl products according to USP chapter “Antibiotics—microbial assays” [38]. This procedure uses the cylinder plate technique, with a minor modification in which sterilized Milli-Q water was used to dilute the vancomycin HCl concentrations (after preparing the stock solution) instead of using monobasic potassium phosphate buffer. On the day of the assay, a stock solution of vancomycin HCl is prepared at a concentration of 1 mg/mL. It is then diluted with sterilized Milli-Q water to achieve a final nominal concentration of 10 μg/mL, matching the median concentration of the standard (labeled S_3_). Concurrently, four additional dilutions are made from the stock solution at concentrations of 2.5 μg/mL, 5 μg/mL, 15 μg/mL, and 20 μg/mL. These dilutions serve as reliable references to accurately evaluate the antibiotic’s efficacy during the experiment (Figure 2 and Figure 3).

Next, the bacterial reference strain *Bacillus subtilis* ATCC 6633 was activated by incubating it on nutrient agar for 24 h at 37 °C. After activation, the bacteria were suspended in 3 mL of sterile saline solution. Then suspension was either spread over two or more agar plates for complete coverage or inoculated into a bottle containing 250 mL of medium no. 32 and kept at 32 °C for five days. This step was crucial to ensure sufficient bacterial growth. (Figure 3) illustrates the preparation steps for the bacterial suspension. After incubation, the *Bacillus* culture was then collected from the plated agar into 50 mL of sterile saline, creating a harvest suspension. The transmittance of this suspension was then measured at 580 nm with a UV-Vis spectrophotometer, targeting about 25% transmittance. This standardizes the volume of the harvest solution added to the seed layer agar. After that, a verification step begins with 1 mL per 100 mL of the inoculation medium No. 8, typically producing adequate zones of inhibition (14–16 mm in diameter) for the median standard concentration (S_3_).

After that, a base layer was prepared by preparing 10 mL of medium no. 32 into Petri-dish plates, which were then solidified to form a smooth base layer of consistent depth. This base layer served as the foundation for the cylinder assay, ensuring a uniform surface for the seed layer. Once the base layer was solidified, the seed layer was prepared. After that, six assay holes were carefully made on the inoculated agar surface (three Petri dishes with two cylindrical holes each), uniformly treated with the median vancomycin HCl concentration (10 μg/mL), plus one hole with a specific concentration from the dilution series. Each set comprised nine plates for a single concentration, adhering to five levels, S_1_–S_5_: 2.5 μg/mL, 5 μg/mL, 15 μg/mL, and 20 μg/mL of vancomycin HCl standard, and an additional set was designated for the sample (U_X_) at 10 μg/mL. Each cylinder hole had an identical volume (180 μL) of antibiotic dilutions. (Figure 3) shows the cylinder plate method for antibiotic assay. The plates were then incubated for 16–18 h at 36 °C, then the diameter of the inhibition zones was measured in millimeters using a digital caliper (Mitutoyo, Kawasaki, Kanagawa, Japan) across the center of a clear inhibition zone. This step was critical for evaluating the efficacy of the tested vancomycin HCl samples against *Bacillus subtilis* ATCC 6633.

### 2.3. Chemical Assay

The HPLC method was developed and validated based on the USP chapter “Vancomycin Hydrochloride for Injection” [35], using an Agilent Technologies-1200 High-Performance Liquid Chromatograph (HPLC) equipped with a Diode Array Detector (DAD). The method was designed to analyze generic vancomycin HCl samples (IV formulations) from multiple manufacturers. Major modifications were made to the USP method [35] to improve resolution, focusing on column type, flow rate, and mobile phase elution mode. These adjustments were critical for enhancing sensitivity, selectivity, and overall analytical effectiveness in the analysis of vancomycin HCl samples. The method was validated according to the International Council for Harmonization ICH Q2(R2), and United States Pharmacopeia (USP) guidelines [40,41]. The validation parameters studied included Linearity, selectivity, limit of quantification (LOQ) and limit of detection (LOD), accuracy (recovery), and precision (inter-day and intra-day precision).

A C18 reversed-phase column (specifications detailed in Table 1) was employed for chromatographic separation. The column’s relatively high backpressure is attributable to its small particle size (3 μm), which enhances chromatographic efficiency and resolution. This design is particularly advantageous for resolving large, complex molecules such as vancomycin HCl. The hydrophobic C18 stationary phase provides excellent retention and separation, yielding sharper peaks and thereby improving the sensitivity and precision of quantification. Collectively, these characteristics render the column well-suited for pharmaceutical analysis, ensuring robust and reproducible separation of vancomycin HCl from potential impurities or matrix components. The column temperature was maintained at 25 °C throughout the analyses.

Chromatographic separation was achieved using an isocratic mobile phase composed of acetonitrile, tetrahydrofuran, and an aqueous solution of triethylamine (0.002% *v*/*v*), in a volumetric ratio of 0.414:0.0142:1. The final mobile phase was adjusted to pH 3.2 with phosphoric acid, then degassed and filtered through a 0.2 μm nylon membrane filter before use. The analysis was performed with an injection volume of 20 μL, a flow rate of 0.5 mL/min, and a column temperature maintained at 25 °C. Detection was carried out at a wavelength (λ) of 280 nm, with a total run time of 30 min per sample. Quantification was based on the integration of the analyte peak area. Detailed chromatographic conditions are summarized in (Table 1).

#### Method Validation

To assess linearity, reference standard vancomycin HCl and sample solutions at five concentrations were analyzed. The results were plotted as vancomycin HCl concentrations versus their peak area responses. A least-squares linear regression was performed using the equation y = mx + c. The reference standard solutions of vancomycin HCl were prepared at five concentration levels: 40, 60, 100, 140, and 200 μg/mL. Each concentration was measured in triplicate. Working standard solutions were made by serial dilution in deionized water from a 200 μg/mL stock solution.

The HPLC-DAD method’s selectivity for vancomycin HCl was evaluated by injecting various concentrations of the sample and reference standard. Then, the vancomycin retention times were recorded. In addition, the sensitivity of the HPLC-DAD method was assessed by LOQ and LOD. The LOQ is based on precision and accuracy at the lowest calibration level, referring to the smallest concentration that can be quantitatively analyzed with acceptable precision and accuracy. It is calculated as: LOQ = 10 (δ/S), where S is the mean of the slopes of the calibration curves and δ is the standard deviation of responses. While the LOD is the smallest concentration or amount of analyte that can be detected but not necessarily quantified, determined by LOD = 3.3 (δ/S).

Precision measures the closeness of analytical results obtained from a series of replicate measurements under the same operating conditions. It reflects random errors and expresses the degree of scatter (closeness of agreement). To evaluate the precision of the method, three replicates of standard solutions at three different concentrations within the calibration range were assayed under similar analytical conditions. These measurements were performed on the same day for repeatability (intra-day) and on two different days for intermediate precision (inter-day). The precision of an analytical procedure is usually expressed as relative standard deviation (%RSD), calculated as: %RSD = (Standard Deviation/Mean) × 100

The accuracy of the analytical method expresses the closeness of agreement between the observed value and the true or expected reference value. It is reported as percentage, calculated using the following equation: recovery% = (Observed value/True Value) × 100.

### 2.4. Data Analysis

#### 2.4.1. Microbiological Assay

Vancomycin antibiotic efficacy was determined by interpolating from a standard curve using a log-transformed linear least-squares fitting approach. The analysis followed defined criteria, emphasizing three key concepts [38].

##### Non-Linearity of Biological Concentration-Response

The relationship between concentration and response is generally non-linear. The method limits the concentration range to ensure results approximate linearity. The assay’s validity requires that the calculated potency lies between 80% and 125% of the presumed concentration used in the sample preparation. If the determined antibiotic potency falls outside this range, it indicates the concentration exceeded the linear range and necessitates adjusting the presumed potency and re-running the assay.

##### Reducing Assay Variability

Conducting multiple independent assay runs enhances the reliability of potency estimates compared to relying on a single extensive assay. Geometric means several independent assays yield a more robust outcome. To accurately evaluate the potency of vancomycin HCl, at least three independent assays are necessary.

##### Sample Data with Linear Regression Analysis

The average zone measurements (standard and sample) across three plates were adjusted for inter-plate differences to determine the potency of unknown materials. A regression line was derived from the adjusted data to estimate the sample’s log concentration. A minimum coefficient of determination (%R^2^), typically no lower than 95% was required, and the final potency was then calculated using the adjusted zone data and the standard curve. The standard curve line was determined following the equation: Z = m × ln(C) + b. Where Z represents the corrected zone measurement, m is the slope of the curve, C denotes the concentration, and b is the intercept. Then, the variation value between plate-to-plate was corrected according to the equation: X_C_ = X_S_ − (X_R_ − P). Where XC represents the corrected standard mean, XS is the original standard mean, XR is the reference mean, and P is the correction point. Then, the sample potency was calculated and determined according to the equation: L_U_ = (U − a)/b. Where a is the intercept of the regression line, and b is the slope of the regression line. Then, the log concentration was converted to the actual concentration by following the equation: C_U_ = e(1.561). After that, to determine the sample potency of the unknown, the antilog of L_U_ was multiplied by the appropriate dilution factor. This value can also be expressed as a percentage of the reference concentration. Lastly, the percentage of the reference concentration was calculated by implanting the equation: Potency (%) = (C_U/_C_References_) × 100.

#### 2.4.2. Chemical Assay

Data analysis for the validation of the developed HPLC-DAD method involved evaluating chromatographic results, with all calculations based on the calibration curve. Validation acceptance criteria were established according to USP and ICH Q2(R2) guidelines [40,41]. The method must be specific to vancomycin HCl, showing no interference from excipients or degradation products at the analyte’s retention time, ensuring accurate identification and quantification. A calibration curve must exhibit R^2^ ≥ 0.95 to confirm linearity. In addition to linearity, the method was evaluated for accuracy and precision across the method’s concentration range to ensure the method’s reliability. Accuracy is acceptable if the mean recovery of vancomycin HCl ranges from 98% to 102%. Precision was evaluated via intra-day (repeatability) and inter-day (Intermediate precision) tests. The %RSD should not exceed 2.0% for intra-day precision and 3.0% for inter-day precision. These criteria ensure that the HPLC-DAD method is robust, reliable, and suitable for quantifying vancomycin HCl in drug products, aligning with pharmaceutical standards.

## 3. Results and Discussion

### 3.1. Microbiological Assay Results

**Figure 4:**
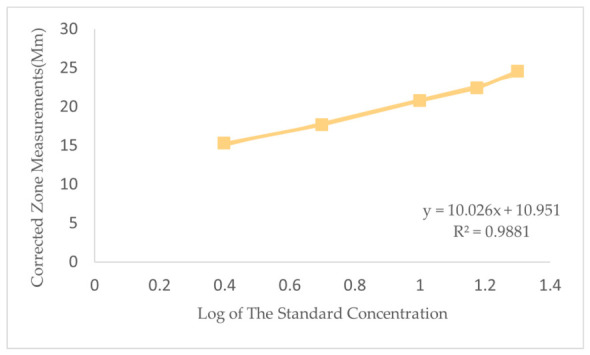
Calibration curve of vancomycin HCl using the microbiological assay. The linear regression of the corrected inhibition zone diameters (mm) plotted against the logarithm of the standard concentrations. Linear regression shows a direct relationship over the specified range, with a correlation coefficient (R^2^) of 0.988. Acceptance criterion: R^2^ ≥ 0.95.

A microbiological assay was conducted to assess the potency of vancomycin HCl using *Bacillus subtilis* ATCC 6633 via the agar diffusion method, as outlined in USP <81> [38]. The findings demonstrated a distinct linear correlation between vancomycin HCl concentrations and inhibitory zone diameters, with the coefficient of determination (R^2^) exceeding 0.98 in all tests, surpassing the statistical acceptance threshold of ≥0.95. This indicates a strong alignment with the logarithmically transformed linear regression model. (Figure 4) shows the Agar diffusion of *Bacillus* subtilis and vancomycin HCl; analyzed using linear fitting of inhibition zone measurements (mm) plotted against the logarithm of the standard concentration. A standard curve was derived from five known standard concentration levels to generate a regression equation, which was then used to interpolate the potency of the samples (U_1_–U_5_).

### 3.2. Chemical Assay Results

**Figure 5:**
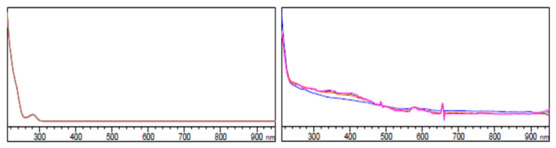
UV–Vis spectrum of vancomycin HCl standard (190–950 nm). The spectrum was recorded using the diluent as a blank. Maximum absorbance was observed at 280 nm, establishing the λ_max_ for Vancomycin HCl and guiding peak area detection and quantification.

The HPLC-DAD method for quantifying vancomycin HCl involved optimization of critical analytical parameters. This process began with wavelength selection, conducted by scanning the vancomycin HCl standard solution from 190 to 950 nm, using the diluent as a blank. The UV-Vis spectrum identified a distinct absorption maximum at 280 nm, which was consequently selected as the detection wavelength (λ_max_) to ensure optimal sensitivity for the quantification of vancomycin HCl in the subsequent HPLC-DAD analyses (Figure 5).

**Figure 6:**
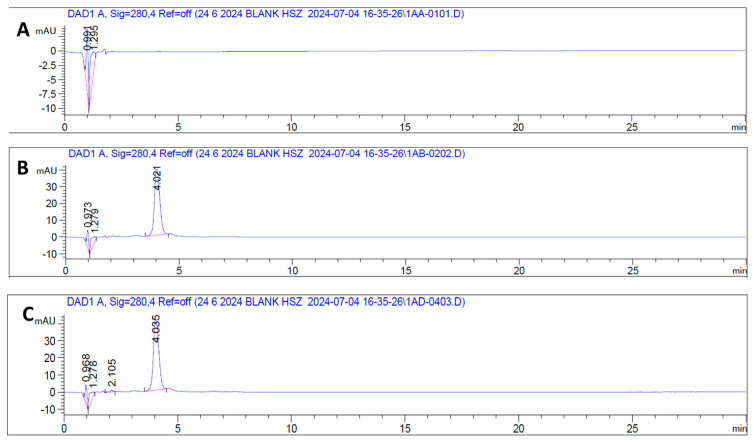
Representative chromatograms obtained via HPLC-DAD at 280 nm. (**A**) Blank sample showing no significant peaks; (**B**) reference standard of vancomycin HCl displaying a distinct peak at approximately 4.0 min; and (**C**) test sample of vancomycin HCl exhibiting a matching peak at the same retention time, confirming analyte identity and method specificity.

**Figure 7:**
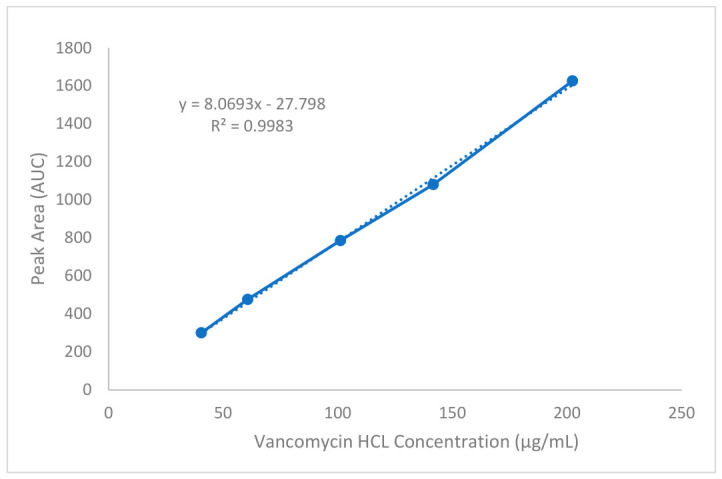
Calibration curve of vancomycin HCl standard measured by HPLC-DAD at 280 nm. The plot demonstrates a strong linear relationship between concentration (40–200 μg/mL) and peak area, with a correlation coefficient of R^2^ = 0.998, indicating the method’s suitability for quantitative analysis.

The optimal chromatographic conditions were then determined by analyzing vancomycin HCl standard (Figure 6B), samples (Figure 6C) of varying concentrations alongside a blank under the same chromatographic conditions. Under the established isocratic mobile phase and flow rate of 0.5 mL/min, vancomycin HCl consistently eluted at a retention time of approximately 4 min, with no interfering peaks observed in the blank chromatogram, as shown in (Figure 6A). Following the determination of the optimal wavelength and retention time, the method’s linear range was established by injecting standard solutions prepared via serial dilution to yield concentrations ranging from 40 to 200 μg/mL. A linear correlation between concentration and peak area was confirmed within this range as shown in (Figure 7).

The method’s range was validated by confirming acceptable precision, accuracy, and linearity for samples containing the analyte of interest within this range. To evaluate the linearity of the HPLC-DAD analytical method, three calibration curves of reference-standard vancomycin HCl were prepared in the range of 40 to 200 μg/mL at five levels 40, 60, 100, 140, and 200 μg/mL, with each level analyzed in triplicate. Working standard solutions were made by serial dilution in deionized water from 200 μg/mL stock solution. The resulting solutions were injected into the HPLC column, yielding peak area responses at approximately 4 min retention time at a flow rate of 0.5 mL/min, measured by the DAD at 280 nm. The linearity data indicated compliance with the validation criteria, as illustrated in (Figure 7) and summarized in (Table 2). The coefficient of determination R^2^ value of 0.998, with the linear relationship described by the equation y = 8.0693x − 27.798. Next, the method’s sensitivity was indicated by LOD and LOQ. Furthermore, (Table 2) shows that the estimated LOD and LOQ values from the calibration curve of 8.7 μg/mL and 26.5 μg/mL, respectively. Subsequently, to further validate the LOQ, six replicates were analyzed for the standard and samples at 40 μg/mL, the results of LOQ indicate a 101.97% and 110.19% recovery value for the standard and sample, respectively. The intra- and inter-day precision were evaluated, where three replicates of standard solutions at three levels (40, 60, and 200 μg/mL) were assayed on the same day, and on two separate days, the relative standard deviation (%RSD) was calculated. (Table 3) shows the repeatability and intermediate precision of the analytical method with RSD% values (0.207–0.134%) and (2.095%), which meet the acceptance criteria with recovery equal to (103.84–100.71%) within-run, and between-run equal to 102.27%. Consequently, this method demonstrates suitable accuracy and precision for the analysis of vancomycin HCl in samples.

### 3.3. Potency Estimation of Generic Products

**Figure 8:**
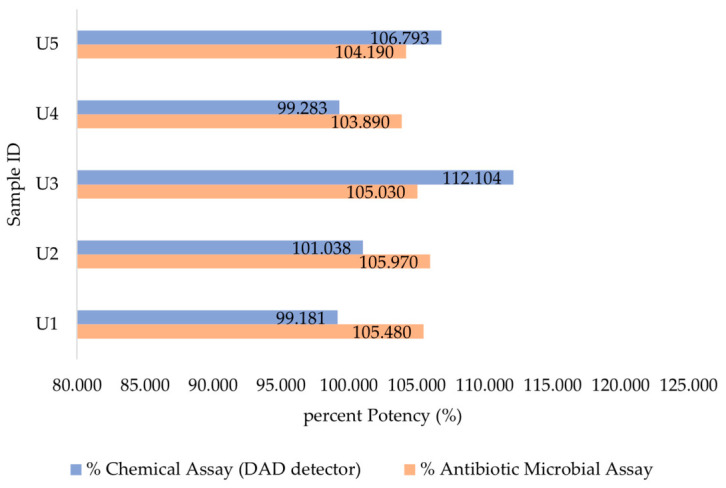
Summary of antibiotic potency (%) of the five tested generic vancomycin HCl products (U_1_–U_5_) available in the Saudi Market. Potency was evaluated using two validated methods: chemical analysis via HPLC-DAD at 280 nm, and microbiological assay, based on inhibition zone measurements. All products demonstrated acceptable potency within specified ranges, supporting their quality and efficacy.

The results of the integrated methods of two techniques chemical and microbiological showed that all the tested vancomycin HCl products met the USP–Pharmacopeia acceptance criteria (80–125%), as shown in (Figure 8). However, the observed variations between the chemical HPLC-DAD and microbiological results indicate a need for further investigation. Because these two methods are not always correlated, they should be conducted in parallel to gain a clear view and a more comprehensive understanding of antibiotic potency by highlighting its biological and chemical properties. (Figure 8) illustrates data from the HPLC-DAD method, along with an antimicrobial assay, highlighting similarities and discrepancies across five generic products (U_1_, U_2_, U_3_, U_4_, and U_5_).

It is also important not to overlook the significance of supporting such studies with clinical studies is crucial, as the pharmaceutical equivalence not always guarantee the therapeutic equivalence. In a pivotal study by Vesga et al. (2010) [16], generics failed to kill *S. aureus* in a neutropenic mouse model, even though they matched the innovator in potency and pharmacokinetics.

The microbial analysis results indicated that the potency of the five tested samples of antibiotics was found to be within the range of 103.89% to 105.97% of the reference potency, as shown in (Figure 8) This reflects meeting biological potency standards and confirming their effectiveness for their intended purpose since the results indicate acceptable in vitro potency according to USP <81>, which defines an adequate range of 80 to 125% of the computed potency. To ensure the validity and accuracy of the assay results, three essential factors were considered: (1) the standard concentration range was precisely selected to ensure linearity within the acceptable range, (2) the calculated sample potency fell within the range of 80% to 125% of the nominal concentration, hence affirming the results’ validity, and (3) test variability was minimized by conducting three independent tests per run, across three separate runs. The geometric mean of potency values across runs and replicates was calculated to enhance the reliability of the final estimate value. The tests exhibited minimal variation, indicating excellent repeatability and substantial reliability.

The DAD consistently demonstrated stable results, with measurements approaching 100% that align with the acceptance criteria, showcasing high reliability and precision, which makes it the preferred choice for assays that demand high accuracy and minimal deviation. Specifically, the HPLC-DAD produced results for samples U_1_, U_2_, U_4_, and U_5_ of 99%, 101%, 99% and 106%, respectively, while sample U_3_ measured 112%. The variations in product potency observed across the different brands tested may be attributed to their manufacturing sources. During the sample preparation, we noticed distinct physical properties among the brands, including differences in shape, color tone, and the solubility time for the powder to fully dissolve into a pure solution. These discrepancies could explain the variations in the results.

Factors contributing to variations in vancomycin quality, especially impurities, should be considered. Fermentation-derived antibiotics like vancomycin tend to generate higher levels of impurities compared to their synthetic counterparts. Due to its complex chemical structure, fermentation-derived vancomycin can produce unwanted byproducts, including analogs, which may lack equivalent pharmacological activity [10,29]. Vesga et al. [16] suggested that differences in the efficacy of generic vancomycin products could be attributed to variable drug levels and elevated levels of impurities such as crystalline degradation product (CDP-1), which can be two to three times higher than in the innovator. CDP-1 can compete with vancomycin for binding sites, thereby reducing its antibacterial activity [15,42]. During the purification, residual impurities—including cell debris and proteins may remain. Additionally, traces of solvents (e.g., ethanol, methanol, and acetone), which are used in manufacturing can persist in the final product, often resulting in reduced activity. Therefore, proper purification and strict quality control is crucial to eliminate impurities and ensure compliance with regulatory standards [15,23,43]. Over time, vancomycin HCl can undergo degradation, producing byproducts that may affect the drug’s purity and functionality [15]. Recent studies suggested that these impurities can act as vancomycin antagonists and may affect their potency and efficacy. This complexity highlights the importance of thorough analytical evaluation to ensure the drug’s potency, safety, and efficacy for its intended use.

Although both chemical and microbiological assays are vital in their respective domains, a combined approach that integrates both methods provides a more thorough scientific and analytical perspective on antibiotics’ quality, safety, and therapeutic efficacy. The study emphasizes the importance of continuous evaluation of generic products to confirm their effectiveness, especially in the treatment of severe infections. Analyzing data on antibiotics available in pharmacies provides valuable insights into their quality and effectiveness while emphasizing the critical role of PMS in ensuring ongoing safety. PMS plays a role in monitoring drug effectiveness and safety after approval, enabling health authorities to detect adverse effects that may not have been evident during pre-marketing trials [44,45]. This surveillance is especially important for generic antibiotics. Although generic drugs must meet stringent standards before approval, real-world data can reveal issues not evident in initial testing [16,46]. Factors such as manufacturing processes and storage conditions can impact the effectiveness of generic drugs once they reach the market. Therefore, continuous monitoring is crucial to ensure that these products remain therapeutically effective, especially in light of rising antibiotic resistance [47]. Moreover, rigorous PMS helps maintain the same quality standards for generic antibiotics as for their branded counterparts. Given the complexity of antibiotic mechanisms of action and their implications for microbial resistance, a robust surveillance system is required that includes feedback from healthcare providers, pharmacists, and patients [47]. Promptly addressing any emerging issues is vital to ensure that marketed antibiotics continue to be safe and effective.

Overall, this study demonstrated that all the tested generic products exhibited adequate biological activity, essential for effectively treating bacterial infections. These findings emphasize the necessity of implementing more targeted programs to evaluate generic antibiotics and ensure their consistent efficacy. Moreover, regular PMS of generics is crucial for safeguarding public health, as it monitors safety, efficacy, and quality long after market release, ensuring sustained effectiveness against bacterial infections. The consistent biological activity observed in these generic products reinforces the importance of rigorous PMS to ensure that they remain safe and effective treatment options, particularly as antibiotic resistance continues to pose a growing threat in clinical practice. Although formal stability studies were not included, as they were beyond the scope of this work, incorporating such assessments in future investigations would provide additional insight into the long-term stability of vancomycin formulations.

## 4. Conclusions

The chemical and microbiological assays emphasize the necessity of utilizing these two methods concurrently to evaluate antibiotic quality and efficacy. The microbiological results confirm that all five generic vancomycin products meet USP <81> standards, indicating their effectiveness against bacterial infections. By combining chemical and microbiological assays, this study provides a comprehensive assessment of antibiotic properties, crucial for combating antibiotic resistance. Moreover, the research highlights the importance of PMS for continuous safety and efficacy, especially given the variability in manufacturing processes. Such ongoing monitoring is essential for maintaining therapeutic effectiveness and protecting public health. These findings emphasize the need for rigorous quality control, the synergy of chemical and microbiological evaluations, and vigilant post-marketing oversight to ensure generic antibiotics remain safe, effective, and ready to address emerging bacterial threats. Moving forward, further research is recommended to explore the impact of manufacturing variations on antibiotic efficacy and to enhance methodologies in post-marketing surveillance.

## Figures and Tables

**Table 1:** Chromatographic Conditions: The parameters used in the HPLC-DAD method development.

Parameter	Specification
Column ^a^	C18, 150 mm × 2.0 mm, 3 µm, 125 Å, Aqua^®^, Phenomenex.
Mobile Phase Compositions ^b^	acetonitrile, tetrahydrofuran, and a triethylamine solution in a ratio of 0.414:0.0142:1 (*v*/*v*/*v*).
Mobile Phase Elution ^c^	Isocratic.
pH ^d^	Mobile phase pH adjusted to 3.2 using phosphoric acid.
Retention Time (Rt) ^e^	About 4 min
Column Temperature	25 °C
Flow Rate ^f^	0.5 mL/min
Injection Volume ^g^	20 µL
Detection wavelength (λ_max_) ^h^	280 nm

^a^ Column details of the chromatographic column, including dimensions, particle size, pore size, and brand. ^b^ Mobile phase composition describes the solvents and their ratios used in the mobile phase. ^c^ Mobile phase elution: Type of elution mode (e.g., isocratic or gradient). ^d^ pH: The acidity or alkalinity of the mobile phase, adjusted to a specific value (here, 3.2) using phosphoric acid to optimize chromatographic separation. ^e^ Rt: Retention time. Time taken for the analyte to elute from the column under specified conditions. ^f^ The volume of mobile phase passing through the column per minute, optimized at 0.5 mL/min to balance resolution and analysis time. ^g^ Volume of sample injected into the chromatograph. ^h^ Wavelength of maximum absorbance used for detection.

**Table 2:** Linearity results for vancomycin HCl: Linearity of standard vancomycin HCl, including estimated LOD and LOQ.

level	Nominal Concentrations (μg/mL)	Rt ^a^ (min)	Mean AUC Responses	Practical Concentrations (μg/mL)	Recovery%
1	40.48	4	300.97	40.74	100.65
2	60.72		476.39	62.48	102.90
3	101.20		786.21	100.88	99.68
4	141.68		1080.97	137.41	96.98
5	202.40		1626.20	204.97	101.27
Mean Recovery%			100.30		
Regression Equation y = mx + b			y = 8.0693x − 27.798		
The coefficient of determination (R^2^)			0.998		
S = Slope (m)			8.069		
Intercept (b)			−27.798		
Concentration Range (μg/mL)			40–200		
δ = standard deviation of response.			21.418		
Estimated LOD ^b^ = 3.3 δ/S. (μg/mL)			8.760		
Estimated LOQ ^c^ = 10 δ/S. (μg/mL)			26.540		

^a^ Rt: Retention time. ^b^ LOD: Limit of detection, the lowest concentration at which the analyte can be reliably detected but not necessarily quantified. Calculated as 3.3 × δ/S, where δ is the standard deviation of response and S is the slope of the calibration curve. ^c^ LOQ: Limit of quantification, the lowest concentration at which the analyte can be quantitatively determined with acceptable precision and accuracy. Calculated as 10 × δ/S.

**Table 3:** Repeatability and intermediate precision. Precision (repeatability and intermediate precision), and accuracy (Within-run, and Between-run) for vancomycin HCl at three concentration levels, according to ICH Q2(R2). Relative standard deviation as a percentage (RSD%); measuring repeatability and intermediate precision.

Nominal Concentrations (μg/mL)	Day1	Day2
	Practical Concentrations (μg/mL)	
40	41.85	37.62
	41.78	37.67
	41.81	37.79
60	62.77	62.77
	62.34	62.87
	62.34	62.90
200	205.35	206.35
	205.92	206.19
	205.77	206.36
RSD% Repeatability (Intra-day)	0.207%	0.134%
RSD% Intermediate precision (Inter-day)	2.095%	
Recovery% (Within-run)	103.84%	100.71%
Recovery% (Between-run)	102.27%	

## Data Availability

The data presented in this study are available upon request from the corresponding author due to privacy reasons.

## Abbreviations

The following abbreviations are used in this manuscript:

HPLC	High Performance Liquid Chromatography
DAD	Diode Array Detector
AUC	The Area Under the Curve
Rt	Retention Time
min	Minute
Avg	Average
RSD	Relative Standard Deviation
SD	Standard Deviation
ICH	International Council for Harmonization
USP	United States Pharmacopeia
Vancomycin HCl	Vancomycin Hydrochloride
IV	Intravenous injection
CC	Calibration Curve
LOD	Limit of Detection
LOQ	Limit of Quantitation
PMS	Post-Marketing Surveillance
